# Effect of Fast High-Irradiance Photo-Polymerization of Resin Composites on the Dentin Bond Strength

**DOI:** 10.3390/ma15217467

**Published:** 2022-10-25

**Authors:** Tobias Steffen, Matej Par, Thomas Attin, Tobias T. Tauböck

**Affiliations:** 1 Clinic of Conservative and Preventive Dentistry, Center for Dental Medicine, University of Zurich, 8032 Zurich, Switzerland; 2 Department of Endodontics and Restorative Dentistry, School of Dental Medicine, University of Zagreb, Gunduliceva 5, 10000 Zagreb, Croatia

**Keywords:** high-irradiance light curing, rapid photo-polymerization, bulk-fill resin composites, micro-tensile bond strength, dentin adhesion, failure analysis

## Abstract

This study investigated the influence of conventional (10 s at 1160 mW/cm^2^) and fast high-irradiance (3 s at 2850 mW/cm^2^) light curing on the micro-tensile bond strength (μTBS) of bulk-fill resin composites bonded to human dentin. Sixty-four extracted human molars were ground to dentin and randomly assigned into eight groups (n = 8 per group). After application of a three-step adhesive system (Optibond FL), four different bulk-fill composites (two sculptable and two flowable composites) were placed. Of these, one sculptable (Tetric PowerFill) and one flowable (Tetric PowerFlow) composite were specifically developed for fast high-irradiance light curing. Each composite was polymerized with the conventional or the fast high-irradiance light-curing protocol. The specimens were cut into dentin-composite sticks, μTBS was determined and failure modes were analyzed. Statistical analysis was performed using *t*-test for independent observations and one-way ANOVA. A statistical difference between the curing protocols was only found for Tetric PowerFlow, where the conventional protocol (23.8 ± 4.2 MPa) led to significantly higher values than the fast high-irradiance light-curing protocol (18.7 ± 3.7 MPa). All other composite materials showed statistically similar values for both polymerization protocols. In conclusion, the use of fast high-irradiation light curing has no negative influence on the μTBS of the investigated high-viscosity bulk-fill composites. However, it may reduce the dentin bond strength of flowable bulk-fill composite.

## 1. Introduction

Resin composites are the most used dental restorative materials for direct restorations [1]. Since the inception of resin composites, shrinkage, and the associated stress development on the adhesive bond during light curing is controversially discussed. Studies have shown that stress development during light curing can lead to marginal gaps or cause fractures when placing too large composite layers [2,3]. Therefore, composite layers should not exceed 2 mm thickness to reduce polymerization shrinkage and allow enough blue light to penetrate the composite material [2,4]. However, the process of placing multiple small composite layers can be challenging in difficult clinical conditions and often time consuming [5].

Creating stable dentin adhesion is challenging due to its water content and smear layer formation [6]. The loss of dentin-composite bond strength can lead to the formation of marginal gaps [7], which can be responsible for hypersensitivity, secondary caries or even the loss of restorations [8].

Resin composite materials [9], adhesive systems [10] and light-curing units [11] are constantly developing. Examples are the development of bulk-fill composites or the steadily increasing irradiances of light-curing units (LCU) in recent years [12]. This leads to acceleration and simplification of many clinical steps.

Manufacturers designed bulk-fill composites to simplify and speed up the placement of resin composite restorations. These materials show considerably higher light transmittance [13,14] and can be placed in increments of 4–5 mm thickness [15,16]. Even in large and deep cavities bulk-filled with such materials, the development of shrinkage stress is lower than in conventional composites [17,18], which can decrease the development of marginal gaps [19]. This behavior is particularly relevant in high C-factor cavities [20] rather than low ones like class IV cavities in anterior direct restorations [21]. Furthermore, the reduced working time results in a lower risk of fluid contamination. Due to their pleasant properties, bulk-fill composites are often used for the restoration of large defects. There are sculptable and flowable bulk-fill composites available. Flowable bulk-fill composites are particularly attractive for the practitioner as they are simple and quick to use. However, due to their reduced filler content, capping with a conventional high-viscosity resin composite material is needed. In contrast to flowable bulk-fill composites, sculptable bulk-fill composites can be placed in occlusal areas without the need of placing a capping composite layer.

To reduce polymerization shrinkage stress of resin composites, different light-curing protocols (soft-start, pulse-delay) have been investigated [22,23,24]. An in vitro study showed that lower light intensity during photoactivation can maintain the gel phase of the resin composite for a longer time. This reduces the shrinkage forces by allowing more viscous flow inside the material [22]. Despite the promising results, this concept was not established clinically since such techniques are technically complicated and time consuming. Furthermore, no significant changes in postoperative sensitivity or marginal integrity were detected with the soft-start light-curing approach [25].

Any time-saving approach is attractive to the practitioner, which has led to the approach of polymerizing resin composites for a shorter time but with higher irradiance. This increased simplicity and efficiency during dental treatments.

LCUs enabling high-irradiance light curing with shorter curing times were developed [26]. Simultaneously, resin composites were designed that polymerize sufficiently at higher irradiances and shorter light-curing times [27]. The latest generation of bulk-fill composites has been specially developed for rapid high-irradiance light curing and is claimed to allow sufficient polymerization in only three seconds [28]. Camphorquinone is a key photoinitiator for the process of polymerization in many resin composites. Other photoinitiators like Ivocerin (Ivoclar Vivadent, Schaan, Liechtenstein) or Lucirin TPO play a predominant role in enabling short curing times with a sufficient depth of cure [29].

The latest generation of bulk-fill composites mentioned above includes a sculptable (Tetric PowerFill) and a flowable (Tetric PowerFlow) resin composite which were specially developed for rapid (3 s) high-irradiance light curing combined with a matching LED-LCU and radiant exitances up to 3000 mW/cm^2^ (Tetric Bluephase PowerCure LED-LCU). However, recent studies have revealed that fast high-irradiance light curing can create high shrinkage stresses within the composite material and at the adhesive dentin–composite interface, which might affect dentin bond strength [3,30].

The aim of this study was therefore to compare the micro-tensile bond strength (μTBS) to dentin of different bulk-fill composites light cured with either a conventional (10 s at 1160 mW/cm^2^) or rapid high-irradiance (3 s at 2850 mW/cm^2^) curing protocol. Four bulk-fill composites were tested. Two of them were specially developed for rapid high-irradiance light curing. The null hypothesis claimed that (I) neither the composite material (II) nor the light-curing protocol used would affect the μTBS.

## 2. Materials and Methods

### 2.1. Composite Materials

**Figure 1:**
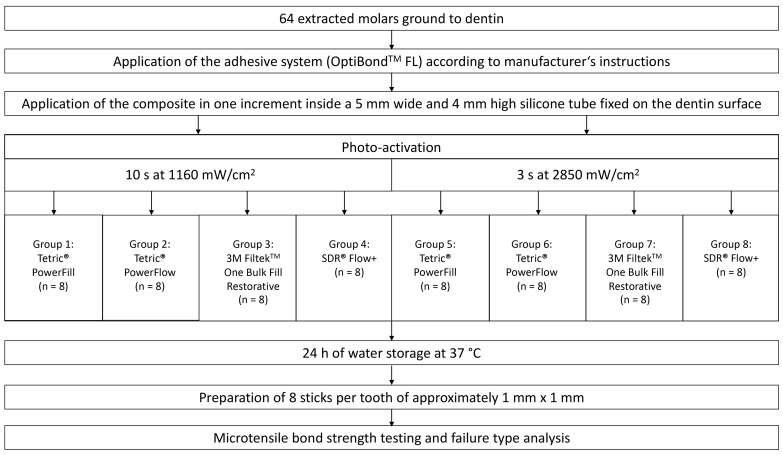
Flowchart of the study.

Four light-curable bulk-fill resin composites were investigated. Two of them were sculptable and two of them were flowable. One sculptable (Tetric PowerFill, Ivoclar Vivadent, Schaan, Liechtenstein) and one flowable (Tetric PowerFlow, Ivoclar Vivadent, Schaan, Liechtenstein) composite were specially developed and approved for high-irradiance light curing. The other sculptable (3M Filtek One Bulk Fill Restorative, 3M, St. Paul, MN, USA) and flowable (SDR Flow +, Dentsply Sirona, Konstanz, Germany) composites have not been specifically developed for high-irradiance light curing. Each composite was photo-activated with the conventional (10 s at 1160 mW/cm^2^) or high-irradiance (3 s at 2850 mW/cm^2^) light-curing protocol. This resulted in eight experimental groups. The schematic experimental setup with the different material groups and light-curing protocols is shown in Figure 1. Table 1 shows the chemical composition and additional information of the resin composite materials used in this study.

### 2.2. Specimen Preparation

For this in vitro study, 64 extracted human molars were collected. The teeth were extracted due to medical indications during normal dental treatment. Before the teeth were collected for the study, patients had to give written informed consent for the further use of the teeth as anonymized biological materials for research purposes. After extraction, the teeth were irreversibly anonymized. The study was thus performed in accordance with the Federal Act on Research involving Human Beings (Human Research Act; article 2, paragraph 2) and authorization from the ethics committee was waived (BASEC-Nr. Req-2021-01153).

Only undamaged and caries-free teeth were used, and biological remains like bone, dental calculus or soft tissue were removed from the teeth. The unrestored teeth were randomly assigned into the eight groups mentioned above (four composites cured with two curing protocols; n = 8 per group). Until their use, the teeth were stored in tap water at 5°C. To facilitate further manipulation, the teeth were fixed on a custom-made carrier (Wenka, Karl Wenger SA, Courgenay, Switzerland) with a light-curable resin (LC Block-Out Resin, Ultradent Products Inc., South Jordan, UT, USA). After fixation on the carrier, the teeth were embedded in self-curing acrylic resin (Paladur, Heraeus Kulzer, Hanau, Germany) which covered two thirds of the tooth roots.

The crowns of the teeth were removed below the deepest point of the fissure with a low-speed precision cutter (IsoMet, Buehler, Lake Bluff, IL, USA) and a diamond coated saw wheel (M0D10, Struers, Birmensdorf, Switzerland; diameter: 102 mm, thickness: 0.3 mm). The surface was then ground from occlusally with 180-grit silicon carbide paper (Buehler-Met II, Buehler, Lake Bluff, IL, USA) by using a polishing machine (Planopol-2, Struers, Ballerup, Denmark) until all enamel remnants were removed. This created a roughening effect similar to that of an 80-μm diamond bur [31]. The polishing machine ran at low speed (150 rpm) with constant water cooling, avoiding any heat development. A stereomicroscope (Stemi 2000, Carl Zeiss, Feldbach, Switzerland) was used to ensure that there were no remnants of enamel on the flat dentin surface and the pulp was not exposed.

### 2.3. Composite Buildup

A three-step adhesive system (OptiBond FL, Kerr, Orange, CA, USA) was applied according to the manufacturer’s instructions. After phosphoric acid etching (Ultra-Etch, Ultradent, South Jordan, UT, USA) for 15 s and rinsing with water for 15 s, the primer was applied for 15 s to the gently air-dried dentin. Then the primer was air-dried until the dentin acquired a shiny appearance. The adhesive was subsequently applied, and light cured at 1160 mW/cm^2^ for 10 s.

The carrier of the specimen was clamped in a custom-made holder which allowed to press a 5 mm wide and 4 mm high silicone tube on the flat dentin surface. This enabled the stable placement of the composite in one increment. The surface was flattened using conventional modelling instruments.

According to their group, the composites were light cured with either low- or high-irradiance. Thus, each composite was cured for 3 s at 2850 mW/cm^2^ (Bluephase PowerCure, Ivoclar Vivadent, Schaan, Liechtenstein; emission wavelength range: 385–515 nm) or for 10 s at 1160 mW/cm^2^. The tip of the light guide locked in the opening of the custom-made holder. This ensured keeping a distance of 1 mm to the composite buildup.

The radiant exitances were periodically controlled using a calibrated dental radiometer (FieldMaxII-TO, Coherent; Santa Clara, CA, USA). The specimens were dark-stored in tap water at 37 °C during 24 h before μTBS testing.

### 2.4. Micro-Tensile Bond Strength Test

The specimens were cut perpendicular in two directions using a water-cooled saw (Accutom-50, Struers, Birmensdorf, Switzerland) and a diamond coated saw wheel (M0D10, Struers, Birmensdorf, Switzerland; diameter: 102 mm, thickness: 0.3 mm). After cutting, the eight most central sticks were marked with a waterproof pen and then sawed off parallel to the occlusal surface of the tooth. The length of the sticks was between 7–8 mm. Sticks were only used when they were exactly square cut and free of enamel. This was controlled with a stereomicroscope (Stemi 2000, Carl Zeiss, Feldbach, Switzerland).

From each tooth, eight dentin-composite sticks were obtained. In total, a group consisted of eight teeth with eight sticks each. This resulted in a total of 64 sticks per group. The sticks were stored in tap water at room temperature to prevent extensive drying of dentin. The edge lengths of the sticks were measured with a digital micrometer (406-250-30, Mitutoyo AG, Urdorf, Switzerland) on the level of the adhesive area. To calculate the adhesive surface of the sticks, the edge lengths were multiplied. The mean adhesive surface of the sticks amounted to 0.921 mm^2^.

The sticks were prepared for μTBS testing according to Armstrong et al. [32]. Both ends were glued into sandblasted (110 μm aluminum oxide, 4.5 bar) μTBS jigs (Wenka, Karl Wenger SA, Courgenay, Switzerland) with cyanoacrylate glue (No. 17330050, Renfert GmbH, Hilzingen, Germany), and mounted in a universal testing machine (Zwick Roell Z010, Ulm, Germany) [33]. Using a load cell of 500 N, a tensile force test was applied with a speed of 1 mm/min until the sticks failed. The load at failure (N) divided by the previously calculated bonding area (mm^2^) of the same stick resulted in the μTBS (MPa). These values were recorded for each stick.

### 2.5. Failure Mode Analysis

The sticks were subjected to a failure mode analysis. Five different failure modes were distinguished: adhesive failures, cohesive failures in the dentin, cohesive failures in the composite, mixed failures, and pre-test failures. The failure modes were observed under a stereomicroscope (Stemi 2000, Carl Zeiss, Feldbach, Switzerland) using 15× magnification.

### 2.6. Statistical Analysis

Power analysis was performed based on a preliminary study to determine the sample size required for identifying a statistically significant difference in bond strength of at least 10% in the comparisons between the two curing protocols. For power analysis the software G*Power (version 3.1, Heinrich-Heine-University of Düsseldorf, Düsseldorf, Germany) was used. Two sticks failed before μTBS testing. According to the protocol of Armstrong et al. [32] the μTBS of these sticks was set to 0 MPa. Descriptive statistics were presented as mean, standard deviation, minimum, median, maximum and 95% CI. Normality of distribution was verified using Kolmogorov-Smirnov and Shapiro-Wilk tests. Statistical comparisons were performed to identify significant differences between groups at an overall significance level of *α* = 0.05. Mean μTBS values for each composite were compared between two curing protocols using *t*-test for independent observations. The comparisons among the composites within a given curing protocol were performed using one-way ANOVA with Tukey’s post-hoc adjustment for multiple comparisons. Statistical analyses were performed using the statistical software SPSS (version 25, IBM; Armonk, NY, USA).

## 3. Results

### 3.1. Micro-Tensile Bond Strength

**Figure 2:**
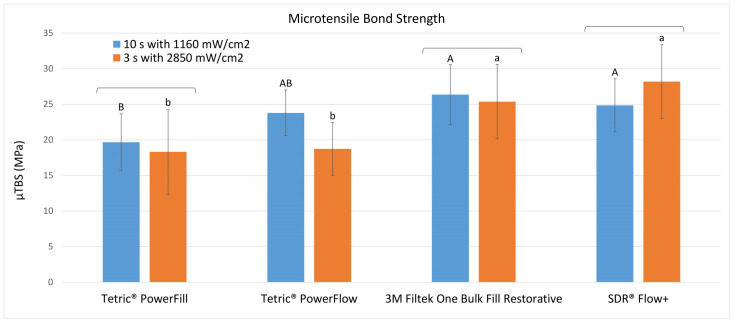
Micro-tensile bond strength (mean ± SD; in MPa) to dentin achieved with the different light-curing protocols. Square brackets above the bars show statistically similar results between the curing protocols (“10 s” vs. “3 s”) within each material. Same uppercase letters indicate statistically similar results among materials for the “10 s” curing protocol. Same lowercase letters indicate statistically similar results among materials for the “3 s” curing protocol.

Table 2 shows descriptive statistics of the μTBS of all groups. The abbreviations of the groups can be seen in Table 1. The results of the statistical evaluation are given in Figure 2.

The only significant difference in dentin bond strength between curing protocols was found for Tetric PowerFlow, where the conventional (10 s at 1160 mW/cm^2^) protocol (G2: 23.8 ± 4.2 MPa) led to significantly higher values than the fast-curing (3 s at 2850 mW/cm^2^) protocol (G6: 18.7 ± 3.7 MPa) (*p* = 0.0002). All other groups showed no significant bond strength differences between the curing protocols.

When comparing the different composites within the conventional (10 s at 1160 mW/cm^2^) protocol, Tetric PowerFill (G1: 19.7 ± 4.0 MPa) attained statistically similar dentin bond strength values as Tetric PowerFlow (G2: 23.8 ± 4.2 MPa), but significantly lower values compared to 3M Filtek One Bulk Fill Restorative (G3: 26.4 ± 6.0 MPa) (*p* = 0.0004) and SDR Flow + (G4: 24.9 ± 5.2 MPa) (*p* = 0.0009).

Within the fast-curing (3 s at 2850 mW/cm^2^) protocol, the composites of the Tetric family (Tetric PowerFill (G5: 18.3 ± 4.2 MPa) and Tetric PowerFlow (G6: 18.7 ± 3.7 MPa)) reached significantly lower dentin bond strength values compared to 3M Filtek One Bulk Fill Restorative (G7: 25.4 ± 3.7 MPa) (*p* = 0.00003 and 0.00008, respectively) and SDR Flow + (G8: 28.2 ± 5.2 MPa) (*p* < 0.000001 for both comparisons).

### 3.2. Failure Mode Analysis

**Figure 3:**
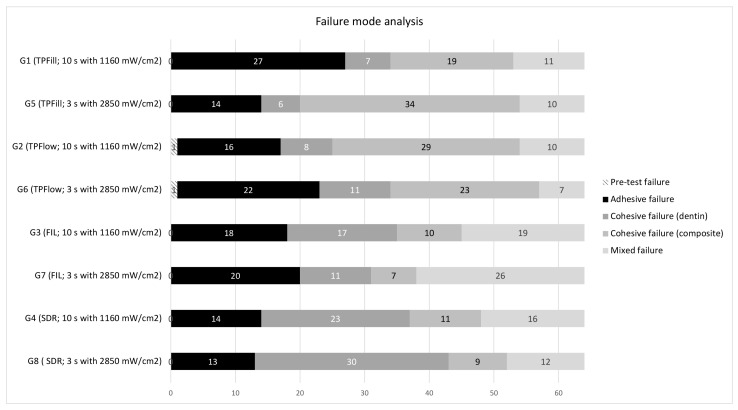
Distribution of failure modes per group. The distribution is given in total numbers.

The distributions of the failure modes of the eight groups are shown in Figure 3. A heterogenous distribution can be seen over all groups. It was found that the failure modes depended more on the material than on the light-curing protocol used. Tetric PowerFlow was the only composite with pre-test failures. 3M Filtek One Bulk Fill Restorative showed more mixed failures than the other composites investigated. Cohesive failures in dentin occurred more frequently with SDR Flow + than with the other composites investigated.

## 4. Discussion

Our findings indicate that Tetric PowerFlow was the only composite with a significant difference in dentin bond strength between light-curing protocols. The μTBS of Tetric PowerFlow was significantly higher with conventional than with high-irradiance light curing. Furthermore, a material dependency of the μTBS was found. Thus, both null hypotheses could be rejected.

Sufficient μTBS is important for direct restorations to prevent the formation of marginal gaps, which have been associated with clinical complications such as secondary caries and postoperative sensitivity [8]. The development of new composites and LED-LCUs has led to continuously increasing material requirements, such as handling high shrinkage stresses [34] or enabling enough blue light to penetrate to the bottom of the composite materials. Previous studies investigating fast-curing composites found that the material itself had a greater impact on various mechanical properties and degree of conversion than the light-curing protocol [35,36,37].

The superior μTBS values of Tetric PowerFlow cured with the conventional protocol were surprising, given that this product was specifically designed for fast high-irradiance light curing. An explanation therefore could be the total energy concept, which makes the polymerization of the composite dependent on the total energy to which it is exposed [38]. This could be relevant because composites require sufficient light energy in a compatible wavelength to achieve appropriate physical properties [39]. The total energies (product of exposure time and light irradiance) of the two light-curing protocols differ as the conventional group was cured with 11.60 J/cm^2^ and the fast-curing group with 8.55 J/cm^2^. It can thus be assumed that the fast-curing protocol leads to less energy arriving at the bottom of the cavity, where the actual bonding occurs. Other studies have shown that a higher total energy also results in better degree of conversion and mechanical properties in deep layers of the composite [40,41]. This may also affect μTBS. In this study, different total energies were used to follow manufacturer’s instructions in light curing. The difference in total energies may be a limitation of this study. Marovic et al. [37] found a lower degree of conversion for Tetric PowerFlow being polymerized with the fast light-curing protocol, which might also contribute to the lower bond strength values observed in this study. Furthermore, Par et al. [42] reported about significantly worse marginal integrity for Tetric PowerFlow polymerized with the fast light-curing protocol.

The μTBS of both sculptable composites was not affected by the light-curing protocol. This finding might be explained by various aspects of the materials. Tetric PowerFill contains an addition fragmentation chain transfer (AFCT) reagent named β-allyl sulfone in the organic matrix, which is intended to delay the gel phase and reduce shrinkage stress [35]. 3M Filtek One Bulk Fill Restorative contains a similar reagent, which is also designed to reduce shrinkage forces [43]. Furthermore, the higher filler content of the sculptable composites limits the mobility of reactive species within the composite during polymerization and can reduce the influence of the light-curing protocol used [13].

Independent of the material or light-curing protocol used, all composites achieved bond strength values between 18.3 and 28.2 MPa. Only Tetric PowerFill and PowerFlow were developed and approved for fast high-irradiance light curing. Nevertheless, the other materials investigated (SDR Flow +; and 3M Filtek One Bulk Fill Restorative) achieved significantly higher dentin bond strength with high-irradiance light curing. Tauböck et al. [44] demonstrated that SDR Flow + develops very low shrinkage forces due to its unique resin composition, which contains modified high-molecular-weight UDMA base monomers. Thus, the adhesive bond is less stressed by shrinkage forces, which could explain the high dentin bond strength values. 3M Filtek One Bulk Fill Restorative performed the best in another dentin bond strength study [45]. The reason might be its modified resin matrix mentioned above, which lowers shrinkage stress as it enables the polymer network to rearrange during polymerization.

The same adhesive system was used for all composites. This combination is approved but does not follow the manufacturer’s recommendations to stay within the same product family, which might have resulted in different bond strengths. Nevertheless, the chosen protocol was used to allow a standardized comparison between the composites.

The failure types were heterogeneously distributed and depended more on the material than the light-curing protocol used. Cohesive failures in composites occurred more frequently with Tetric PowerFlow and PowerFill. Thus, the adhesive bond was stronger than the cohesion of the composites itself. An explanation for this could be the rapid onset of opacity during light curing, which was reported by Marovic et al. [37]. With the rapid onset of opacity of the composite, the light supply for the deep composite layers is restricted, which could explain the inferior mechanical properties. On the other hand, cohesive failures in dentin and mixed failures were the predominant failure modes of SDR Flow + and 3M Filtek One Bulk Fill Restorative, respectively.

A limitation of this study is that only the μTBS was investigated. In the patient’s mouth, not only tensile but also shear and compression forces are exerted on the filling [46]. These forces may compromise the adhesive bond differently. To draw conclusions about the clinical situation, further studies investigating the influence of these different kinds of forces should be conducted. Since dentin development changes over time [47], the tooth sample collection, which was not based on a specific age group, might represent another limitation of the present study. Finally, the μTBS tests were carried out 24 h after the composite restoration was placed and thus the results only provide information about the short-term adhesive bond. Further studies are needed to draw conclusions about the long-term adhesive bond.

Flowable bulk-fill composites have been a breakthrough on the dental market because they are quick and easy to use. Clinicians are used to placing large quantities of flowable bulk-fill composite materials into teeth, which then need to be capped with a sculptable composite. However, rapid high-irradiation light curing can reduce the dentin bond strength of flowable bulk-fill composite (Tetric PowerFlow).

## 5. Conclusions

The results of this in vitro study show that rapid high-irradiation light curing has no negative influence on the μTBS of the investigated high-viscosity bulk-fill composites. However, rapid high-irradiation light curing can reduce the dentin bond strength of flowable bulk-fill composites (Tetric PowerFlow). Further studies are needed to verify the results and to determine other factors influencing μTBS in the long term.

## Figures and Tables

**Table 1:** Manufacturers’ information about the resin composite materials used in this study.

Composite Viscosity	Composite Name (Abbreviation)	Filler Content (wt%/vol%)	Resin Matrix	Photoinitiator	Manufacturer	Shade/LOT no.
Flowable	Tetric PowerFlow (TPFlow)	68/46	Bis-GMA, Bis-EMA, UDMA	CQ/amine, Ivocerin	Ivoclar Vivadent, Schaan, Liechtenstein	IV A/Z02D5S
	SDR Flow + (SDR)	71/47	Modified UDMA, Bis-GMA, TEGDMA	CQ	Dentsply Sirona, Konstanz, Germany	Universal/00070690
Sculptable	Tetric PowerFill (TPFill)	77/54	Bis-GMA, Bis-EMA, UDMA, propoxylated bisphenol A dimethacrylate, DCP, β-allyl sulfone AFCT agent	CQ/amine, Ivocerin, Lucirin TPO	Ivoclar Vivadent, Schaan, Liechtenstein	IV A/Z02H66
	3M Filtek One Bulk Fill Restorative (FIL)	77/59	UDMA, aromatic UDMA, DDDMA, proprietary AFM	CQ/amine	3M, St. Paul, MN, USA	A3/NE28748

Bis-GMA: bisphenol-A-glycidyldimethacrylate, Bis-EMA: ethoxylated bisphenol-A-dimethacrylate, UDMA: urethane dimethacrylate, TEGDMA: triethylene glycol dimethacrylate, DDDMA: 1, 12-dodecanediol dimethacrylate, AFM: addition fragmentation monomer, DCP: tricyclodecane-dimethanol dimethacrylate, AFCT: addition-fragmentation chain transfer, CQ: camphorquinone, TPO: 2,4,6-trimethylbenzoyldiphenylphosphine oxide.

**Table 2:** Descriptive statistics of the micro-tensile bond strength (MPa) of all groups.

Group (G)	Mean	SD	Min.	Median	Max.	95% CI
G1 (TPFill; 10 s at 1160 mW/cm^2^)	19.7	4.0	6.2	20.3	40.7	17.8–21.5
G5 (TPFill; 3 s at 2850 mW/cm^2^)	18.3	3.2	4.3	18.5	17.6	16.6–20.0
G2 (TPFlow; 10 s at 1160 mW/cm^2^)	23.8	4.2	0.0	24.8	34.8	21.9–25.7
G6 (TPFlow; 3 s at 2850 mW/cm^2^)	18.7	3.7	0.0	19.3	17.9	17.0–20.5
G3 (FIL; 10 s at 1160 mW/cm^2^)	26.4	6.0	2.1	27.8	53.8	23.7–29.0
G7 (FIL; 3 s at 2850 mW/cm^2^)	25.4	3.7	3.0	24.9	23.9	23.0–27.8
G4 (SDR; 10 s at 1160 mW/cm^2^)	24.9	5.2	4.4	24.1	51.3	22.2–27.6
G8 (SDR; 3 s at 2850 mW/cm^2^)	28.2	5.2	5.4	28.3	28.4	25.7–30.7

## Data Availability

The datasets generated during and/or analyzed during the current study are available from the corresponding author on reasonable request.
